# Effect of a portion-size default nudge on meat consumption and diner satisfaction: controlled experiments in Stanford University dining halls

**DOI:** 10.1186/s12889-025-22495-9

**Published:** 2025-04-16

**Authors:** A. Voşki, M. Braginsky, A. Zhang, J. Bertoldo, S. Egan, L. A. Levig, M. Mueller Ihrig, M. B. Mathur

**Affiliations:** 1 Emmett Interdisciplinary Program in Environment and Resources (E-IPER), Stanford Doerr School of Sustainability, Stanford University, 473 Via Ortega, Y2E2 Building, Suite 227, Stanford, CA 94305 USA; 2 Department of Psychology, Stanford University, Stanford, USA; 3 Quantitative Sciences Unit, School of Medicine, Stanford University, Stanford, USA; 4 Stanford Dining, Hospitality and Auxiliaries, Residential and Dining Enterprises, Stanford University, Stanford, CA USA; 5 Stanford Food Institute, Stanford University, Stanford, CA USA

**Keywords:** Default nudge Choice-architecture, Behavior intervention, Portion size, Meat consumption

## Abstract

Reducing meat consumption, especially in high-intake countries such as the United States, is crucial in mitigating the climate and biodiversity crises and improving public health and animal welfare. Choice-architecture interventions or nudges in the food domain, such as choice defaults (e.g., reduced default portion sizes), can be powerful levers of behavior change. However, evidence remains limited in large-scale, real-life settings, and little is known about potential effects on diner satisfaction and backfiring effects that reduce or even reverse the desired behavior. These uncertainties have posed substantial barriers to scalability and wider adoption by the food service industry. In our single-blinded, quasi-experimental, pre-registered field interventions in Stanford University dining halls with staff-served portions, a 25% reduction in the serving spoon size (Study 1, 24 days, 364 diners, made-to-order burritos) produced a non-significant trend of 18% less meat served per day without reducing overall diner satisfaction (*p* = 0.059, d = 0.64) but with a wide CI that included the null (- 49.2, 1.07). A more substantial 50% reduction in serving spoon size (Study 2, 29 days, 1802 diners, varying menu items) did not reduce the amount of meat served (*p* = 0.60, d = 0.20), triggered backfiring effects, and significantly decreased diner satisfaction. Combining the two studies, the intervention did not significantly reduce meat consumption. While the trends in our findings are consistent with the ‘norm range model’—i.e., that moderate portion reductions may decrease intake but drastic reductions may prompt compensatory eating—key differences and contextual nuances between the two studies help explain the mixed results. Future studies on the ‘norm range’ of default portion size nudges to reduce meat consumption across different menu items and food service models is suggested to increase our understanding of effective and scalable interventions that facilitate collective shifts towards more sustainable dietary behaviors.

## Introduction

The rapidly intensifying climate and biodiversity crises are causing significant negative impacts on human and planetary health [1, 2]. Food production is the primary driver of accelerating biodiversity loss [3] and a key contributor to climate change, accounting for ~ one-third of global anthropogenic greenhouse gas emissions [4]. Food production and consumption also play a significant role in public health, with poor diets contributing to ~ 11 million annual human deaths globally as a leading cause of morbidity and mortality [5]. Furthermore, industrial-scale animal agriculture results in severe and widespread animal suffering [6–9]. Animal-based food production and consumption disproportionately contribute to these issues; red meat and dairy products are most detrimental to human and planetary health, while chicken, fish, and eggs to animal welfare [8, 10].

More specifically, red and processed meat products are known to increase the risk of several major chronic diseases, such as diabetes, cardiovascular disease, stroke, and certain types of cancers, e.g., colorectal cancer [11–14]. The World Health Organization (WHO) classifies red meat as ‘probably carcinogenic to humans’ and processed meat as ‘carcinogenic to humans’ [15]. The United States (U.S.) leads the Global North and ranks second globally in per capita meat consumption with ~ 347 g/day [16], more than triple the government recommended ~ 105 g/day serving of meat, poultry, and eggs, and significantly exceeding the healthy intake levels set in the 2020–2025 Dietary Guidelines for Americans [17]. Growing consensus supports a shift from animal-based to plant-based food consumption, particularly in countries such as the U.S., to promote human, environmental, and animal welfare [18–20]. However, changing the food habits and dietary preferences of millions is challenging, necessitating more attention and research on this issue [21]. Beyond fixed traits, dietary change is influenced by complex factors including cultural and social context, habits, taste preferences, familiarity, resources, and cooking skills [22–25].

Effective solutions must target individual behavior change and account for these interrelated complex factors while remaining feasible for large-scale and cost-effective societal implementation. Choice-architecture interventions, i.e., nudges or nudging [26], “change behavior by (re)designing the physical, social, or psychological environment in which people make decisions while preserving their freedom of choice” [27]. Nudging interventions that target more automatic or habitual behaviors by altering the decision structure—e.g., changing default choices or the range of options—tend to be more effective, likely due to minimizing the cognitive load and individual differences in goals and values [27–29]. The choice default—pre-setting a specific option that does not require the individual to make an active choice—can be a powerful lever of behavior change. For example, implementing a vegetarian default option at conference lunches significantly increased the vegetarian choice to over 80% of attendees in three randomized controlled field experiments [30]. As eating habits are particularly vulnerable to failures in self-regulation [18], nudges in the food domain show exceptional promise with effect sizes up to 2.5 times larger than in other domains, such as pro-environmental behaviors or health [27].

Systematic reviews indicate that nudges, particularly choice defaults [31], can effectively reduce animal-based food consumption [32–34]. Changing default portion sizes, such as reducing the meat portion, shows considerable potential [27, 32, 34, 35]. Meta-analyses in the health field robustly substantiate the ‘portion size’ effect across a range of individual and environmental contexts [36, 37]. While this effect has been demonstrated in real-life restaurant settings, e.g., a larger portion size led to increased consumption of a pasta dish [38], field-based research on reducing the meat portion size remains limited.

To date, a laboratory experiment [39], and three real-life field experiments in the food industry [40–42], have tested the effect of reduced meat portion sizes on meat consumption. In an American laboratory study, reducing the meat portion size by 13.5% or 33.5% significantly reduced participants’ meat consumption—proportionally corresponding to their respective condition—and neither increased hunger nor decreased fullness [39]. In a month-long Belgian food store experiment, offering reduced-size meat sausages (− 17% or 125 g; − 33% or 100 g) alongside the default regular portion (150 g) significantly increased consumer purchases of the two smaller options, resulting in a reduction of the total volume of meat sold [40]. A Dutch three-restaurant study showed that a 12.5% reduction in meat portion size significantly lowered meat consumption without affecting overall diner satisfaction, despite a significant decrease in main dish satisfaction [41]. Finally, four Dutch restaurant experiments found that reduced-size meat portions consistently reduced meat consumption by 12–34% while maintaining high diner satisfaction [42]. Importantly, these effects held across different types of dining contexts—i.e., six company canteens and a self-service, an à-la-carte, and a buffet restaurant—substantiating feasibility and scalability in a variety of food service settings.

While these three real-life experiments demonstrated promise with plated meals in retail and restaurant settings, more field-based yet rigorous experimental evidence is needed on diner satisfaction and potential backfiring effects. Approximately 15% of interventions are likely to backfire or create a backlash, i.e., reduce or even reverse the desired behavior [27]. This is likely due to nudges having heterogeneous treatment effects partly explained by psychological reactance theory [43]. Indeed, according to the ‘norm range model’, portion size reductions closer to the lower end of the perceived normal range may successfully decrease energy intake, but more drastic reductions likely prompt compensatory consumption [44]. While the effect sizes of nudges have been debated due to publication bias [27, 45], meta-analyses accounting for this bias substantiate the effectiveness of choice default interventions, albeit with considerable variation depending on the underlying nature and number of mechanisms activated [46].

Given ongoing research on effective ‘norm range’ boundaries and uncertainties in different food service settings, diner satisfaction, and backfiring effects, this study assessed staff-served, made-to-order entrees dispensed with serving utensils. This setup is common among major American chain restaurants (e.g., Chipotle, Subway, poke bowl bars) and in various campus dining, workplace, and K-12 school cafeteria settings. Furthermore, controlling the portion with the serving utensil has been shown to effectively promote consumption changes [47, 48]. We therefore tested the effectiveness of a nudge intervention that changed (i.e., reduced) the default size of the serving utensil that was used by staff to serve individual meat portions. While the amount of meat served was a proxy for meat consumption, professional kitchen staff—trained to provide a single level spoon of meat by default—were responsible for the food service. This ensured that the reduced serving utensil size corresponded to the reduced default portion size.

## Methods

We aimed to test the effectiveness of this default portion-size nudge intervention at two campus dining halls at Stanford University. The research project was carried out through our lab’s partnership with chefs and staff at Stanford’s Residential and Dining Enterprises and subject matter experts from the Stanford Food Institute. We used a blinded, between-subjects quasi-experimental study design, wherein participants—diners at the dining halls—were not specifically made aware of the study and therefore the week’s treatment condition. Treatment conditions—control vs. intervention weeks—were equally balanced. Following a two-week pilot study in May 2022, for Study 1, we conducted six weeks of data collection from October 3—November 11, 2022, at Dining Hall 1 with a 25% serving spoon size reduction. For Study 2, we conducted six weeks of data collection from April 10—May 19, 2023, at Dining Hall 2 with a 50% serving spoon size reduction.

Both studies ran during the middle of the university’s 10-week quarter to exclude exam periods and breaks. Both dining halls are located on the same campus but likely serve different, mostly undergraduate, diners given the university’s residential neighborhood system; Dining Hall 1 is centrally located near classrooms, while Dining Hall 2 is peripheral and closer to student residence halls. Both operate on a swipe-in basis, meaning one card swipe at a pre-determined, standardized cost gives diners unlimited all-you-care-to-eat dining access until the end of the meal period.

In both studies, there was only one staff-served food station—the target intervention station—that offered a single type (beef, pork, chicken, or fish) of scoopable meat per day as the only meat ingredient in the lunch station’s overall dish (e.g., ‘al pastor’ pork at “Cardinal Sage” in Study 1’s burritos and tuna fish at “Fireside” in Study 2’s sandwiches). These are known as mixed meat dishes in the behavioral food science literature [49]. This intervention station alternated between treatment conditions. All other non-intervention (“Core Menu”) stations, where diners self-served other meat—e.g., whole meat cuts; known as meat-centered dishes in the literature—were used as an additional, non-strict “control” to assess backfiring effects at other stations. Additionally, the intervention was only implemented on weekdays, due to research staff availability, and at lunch to assess whether the intervention caused backfiring effects at dinner (i.e., diners eating more meat at a subsequent meal due to having eaten less at lunch).

The data collection procedures and planned analyses for both studies were pre-registered via the Open Science Framework (https://osf.io/2hy74/registrations), with all code and data available at https://osf.io/2hy74/. Study 2 was pre-registered after the analysis of Study 1 was complete but prior to Study 2’s data collection. The one minor deviation from the pre-registered analysis plan is disclosed below. This study received approval from Stanford University’s Institutional Review Board (Protocol: 66713) following all guidelines for research with human subjects.

### Study 1: 25% spoon-size reduction at a burrito bar

**Fig. 1:**
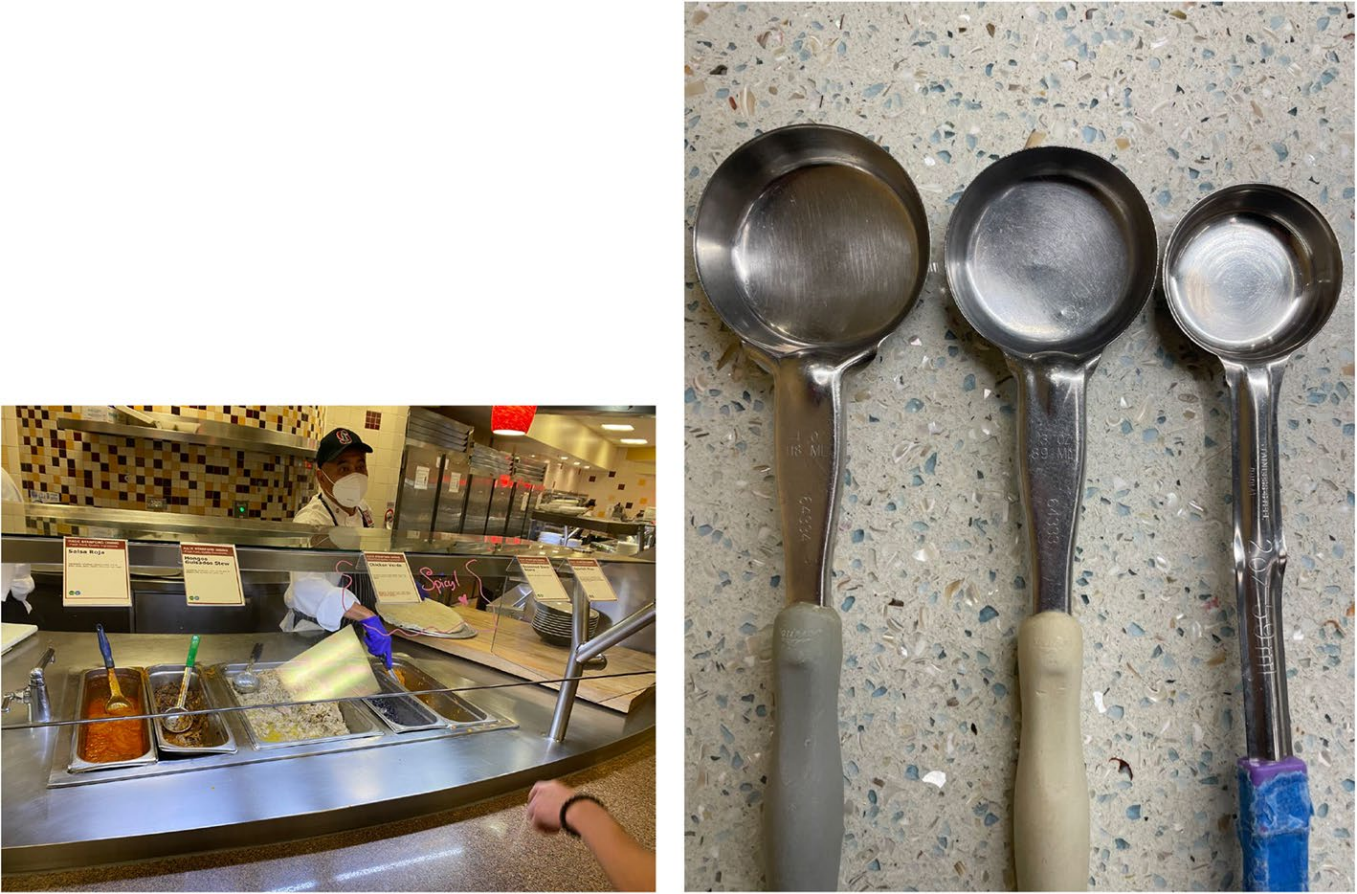
Photographs from the study location showing (**a**) the intervention station in Study 1, and (**b**) a side-by-side comparison of the three serving spoon sizes from left to right: 4 oz, 3 oz, and 2 oz

Dining Hall 1 was chosen as the pilot and Study 1 site because it already had a popular staff-served, made-to-order food station (“Cardinal Sage” burrito bar) that met the intervention’s requirements. Besides the daily choice of one type of scoopable meat, diners could customize their burrito with vegetarian and/or plant-based (i.e., vegan) ingredients, such as rice, beans, vegetables, and condiments such as salsa (Fig. 1a).

#### Intervention and control conditions

Prior to this study, the intervention station standardly used 4 oz serving spoons. Therefore, during control weeks, the same 4 oz spoons were used to scoop the meat at the station, while 3 oz spoons—25% reduction—were used during intervention weeks. The visual size difference between the two spoon sizes was subtle (Fig. 1b, left and middle spoons). Consistent with choice default nudges, serving staff at the intervention station were instructed to provide an additional, i.e., beyond the standard one-scoop, serving of meat only to diners who explicitly requested it, in the same manner they normally would were the study not taking place, and to withhold any study-related information from diners. Adherence to the study instructions (i.e., treatment fidelity) was ensured by the first author’s frequent presence and oversight at the intervention station. Furthermore, at the control stations, the available dish types differed daily as well as between lunch and dinner. Before the self-service dinner period, research assistants and kitchen staff ensured serving spoons at all stations were switched back to the control size.

Data collection consisted of three stages: pilot (2 weeks; 5 control/5 intervention meals), main study with staff-served meat portions (4 weeks; 23 control/15 intervention meals), and a secondary study with self-served meat portions (2 weeks; 10 control/8 intervention meals). The variations in sample sizes reflected unforeseen data collection challenges (e.g., power outages from storms). The secondary study was excluded from our analyses as it was not pre-registered and had a small sample size. The pilot study was also excluded from analyses as its purpose was to optimize our data collection protocols without interfering with the dining hall’s busy operations during peak hours. The control (Weeks 1 and 3) and intervention (Weeks 2 and 4) conditions alternated in an equally balanced, pre-planned manner. Data collection coincided with Halloween, but from previous experience, this holiday was not expected to significantly impact diner attendance patterns.

#### Measures

Undergraduate research assistants, the first author, and kitchen staff measured and double checked the total weight of meat at each food station (both intervention and control stations) before and after each meal period (lunch and dinner). As such, the main measure of intervention effectiveness was the total amount of meat served (i.e. difference between before and after weight) at the intervention vs. control stations. A secondary measure of the amount of meat served was per-diner consumption, meaning the total meat served divided by the total number of diners eating at the dining hall during the meal period. The total number of diners was obtained from the card swipes, which are required to enter the dining hall. We also measured potential backfiring effects as described in the previous section. The dataset thus consists of daily measures for each food station (intervention vs. control), each mealtime (lunch vs. dinner), and treatment condition (control vs. intervention week).

We also administered an online questionnaire using the Qualtrics platform to measure whether the intervention affected diner satisfaction. Participants were recruited via flyers placed throughout the area, including on the dining tables, on data collection days only and were compensated with $5 gift cards. The questionnaire asked whether the participant ate from the intervention station that day, and if so, whether they consumed meat.^1^ Participants then separately rated (on a 0–100 sliding scale) how satisfied, hungry, and full they felt at the time of survey completion (0 being 0% satisfied and 100 being 100% satisfied). Diners also reported demographic traits and dietary habits.

### Study 2: 50% spoon-size reduction with varying menu items

**Fig. 2:**
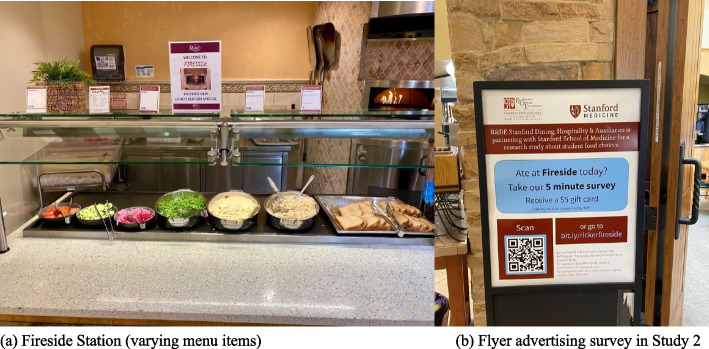
Photographs from the study location showing (**a**) the intervention station in Study 2, and (**b**) the flyer at the entrance door advertising the online survey to diners in Study 2

To test whether further reductions in the amount of meat served could be achieved with an even smaller serving spoon size and in a more heterogeneous setting (i.e., more diverse menu items than made-to-order burritos only), we conducted Study 2 in Dining Hall 2. As in Study 1, there was only one staff-served, made-to-order food station (“Fireside” lunch special) that served varying daily dishes with scoopable meat, e.g., tuna sandwiches and chicken souvlaki (Fig. 2).

#### Intervention and control conditions

During control weeks, the same standardly used 4 oz spoons were used to scoop the meat at the intervention lunch station, while 2 oz spoons—50% reduction—were used during intervention weeks. The visual size difference between the two spoon sizes was much more salient than in Study 1 (Fig. 1b, middle and right spoons). Serving staff received the same training about the serving protocol and study information sharing as in Study 1. Similarly, at the control stations, the available dish types differed daily as well as between lunch and dinner. Before the self-service dinner period, kitchen staff ensured serving spoons at all stations were switched back to the control size.

In total, 29 days of data were collected (6 weeks; 14 control/15 intervention meals). The control (Weeks 1, 3, 4) and intervention (Weeks 2, 5, 6) conditions alternated in a balanced, pre-planned manner. Data collection was not planned for the 30th day due to a scheduled all-day event with a special menu that prevented study implementation.

#### Measures

**Fig. 3:**
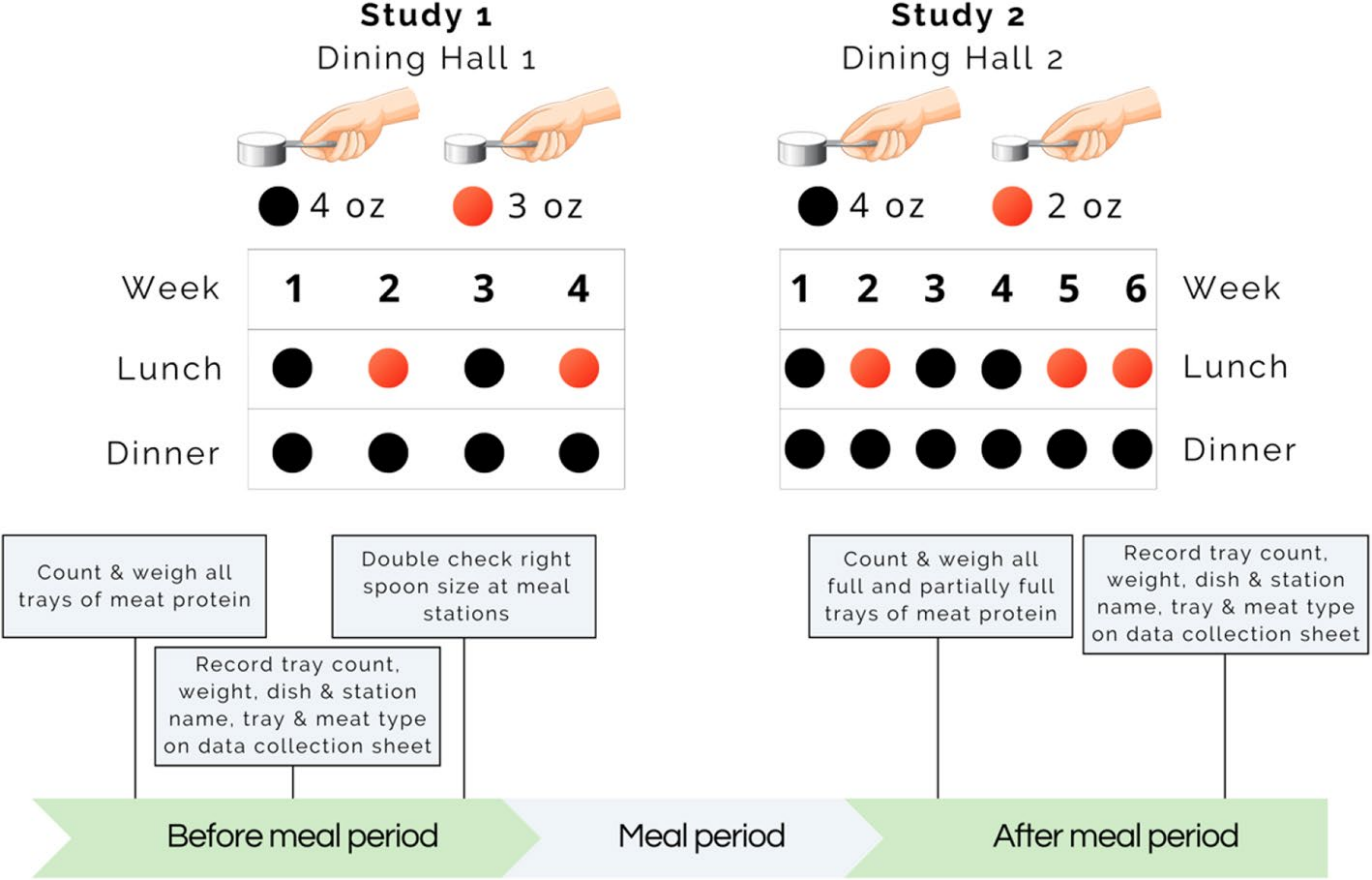
Study timeline and procedure to measure intervention effectiveness as well as potential backfiring effects at non-intervention stations during lunch and at all stations during dinner. Black dots indicate control weeks (4 oz serving spoons used by staff); red dots indicate intervention weeks (3 oz spoons in Study 1 and 2 oz spoons in Study 2)

As in Study 1, kitchen staff measured and double checked the total weight of meat at each food station (both intervention and control stations) before and after each meal period (lunch and dinner). The main measure of intervention effectiveness was the total amount of meat served at the intervention vs. control stations. Secondary measures constituted per-diner consumption, potential backfiring effects, and meat type as a potential interaction term to determine whether the intervention’s effect varied across different meat types. The same survey recruitment approach and online questionnaire used Study 1 were administered (Fig. 3).

### Data analysis

We tested the following pre-registered hypotheses^2^:

**Table:** 

H1		Meat consumption: Less meat will be served at the intervention station (at lunch) during intervention weeks than during control weeksAQ
H2	a^a^	Backfiring effects (other stations at lunch): Considering the entire dining hall (non-intervention stations), the amount of meat served will not be higher during intervention weeks vs. control weeks
	b	Backfiring effects (at dinner): Considering dinner times (when the intervention never takes place), the amount of meat served will not be higher during intervention weeks vs. control weeks
H3		Secondary analysis (diner satisfaction) and related measures (hunger and fullness) will be comparable during invention weeks and control weeks
H4		Secondary analysis (meat types): For each above hypothesis, we are also interested in whether the effect varies across different meat types

^a^For Study 1, only H2b was pre-registered and as such only that analysis was originally conducted, while for Study 2 both H2a and H2b were pre-registered. However, we ran a post hoc analysis for H2a in Study 1 and report the results in footnote #4

Data were transcribed from data collection sheets submitted by kitchen staff and research assistants into Microsoft Excel. All meat dishes (scoopable meat for the intervention station and other meat for the non-intervention stations) only contained a single meat type and were always stored and served from industry standard metal trays. As these were varying tray sizes, the weight of each tray was subtracted from the final recorded weight to accurately capture the true amount of meat served and, by proxy, consumed.

For Study 1, six days had missing data due to unforeseen changes in research assistant schedules, kitchen staffing shortages, and power outages in the dining hall. Since this appears to be missingness completely at random (MCAR), we performed complete-case analysis. For Study 2, there were no missing data. In both studies, only survey responses that were marked as complete, that indicated having eaten from the intervention station, and that were submitted within three hours of the end of the lunch period, were analyzed. All analyses were conducted in R [50], with figures produced using the ggplot2 package [51], and *p* < 0.05 indicating statistical significance. The sample size was determined based on logistical constraints, specifically the maximum feasible study duration within one academic quarter. Multilinear regressions, coefficient tests, and analysis of variance (ANOVA) were employed for analysis. We aggregated the results of Study 1 and Study 2 by refitting the primary analysis models with a fixed effect of study,^3^ as a form of internal meta-analysis using individual participant data.

## Results

### Study 1: 25% spoon-size reduction at a burrito bar

#### Demographics and sample size

A total of 24 days of data were collected for the weight measures, with 12 days (50%) under the intervention condition. The total number of diners eating at the dining hall was *n* = 13729 during lunch and *n* = 6597 during dinner. The flyers specifically prompted survey responses from diners who ate at the intervention station (Fig. 2b); as such, the resulting sample of *n* = 364 (219 control/145 intervention condition) represented 2.65% of all diners during the lunch period. More specifically, the weekly breakdown for survey responses was 151 (Week 1, control), 96 (Week 2, intervention), 68 (Week 3, control), and 49 (Week 4, intervention). Survey respondent demographics and dietary habits are presented in Table 1.

**Table 1:** Survey respondent demographic traits and dietary habits in Study 1 and Study 2. Chi-square tests were used to determine that the conditions did not differ significantly based on these six variables

		**Study 1**	**Study 1 – Test for difference between conditions**	**Study 2**	**Study 2 – Test for difference between conditions**
**Gender**	Female	56%	*p* = 0.21	53%	*p* = 0.22
	Male	41%		42%	
	Non-binary	1.5%		1%	
	Declined to answer	1.5%		4%	
**Race**	Asian or Pacific Islander	38%	*p* = 0.24	39%	*p* = 0.23
	White or Caucasian	26%		30%	
	Hispanic or Latino	15%		6%	
	Black or African American	3%		5%	
	Mixed	12%		13%	
	Declined to answer	6%		6.5%	
**College class**	Undergraduate student	91%	*p* = 0.22	94%	*p* = 0.22
	Graduate student	7%		2%	
	Other	2%		4%	
**Diet**	Omnivore	62%	*p* = 0.22	75%	*p* = 0.22
	Flexitarian	22%		16%	
	Vegetarian	7%		5%	
	Vegan	5%		2%	
	Pescatarian	3%		3%	
**Weekly meat consumption frequency**	0 times	16%	*p* = 0.21	26%	*p* = 0.22
	1–7 times	33%		49%	
	8–14 times	39%		13%	
	15–21 times	11%		3%	
**Weekly fish consumption frequency**	0 times	32%	*p* = 0.21	53%	*p* = 0.22
	1–7 times	59%		27%	
	8–14 times	5%		~ 1%	
	15–21 times	0%		~ 1%	

#### Meat consumption (H1)

**Fig. 4:**
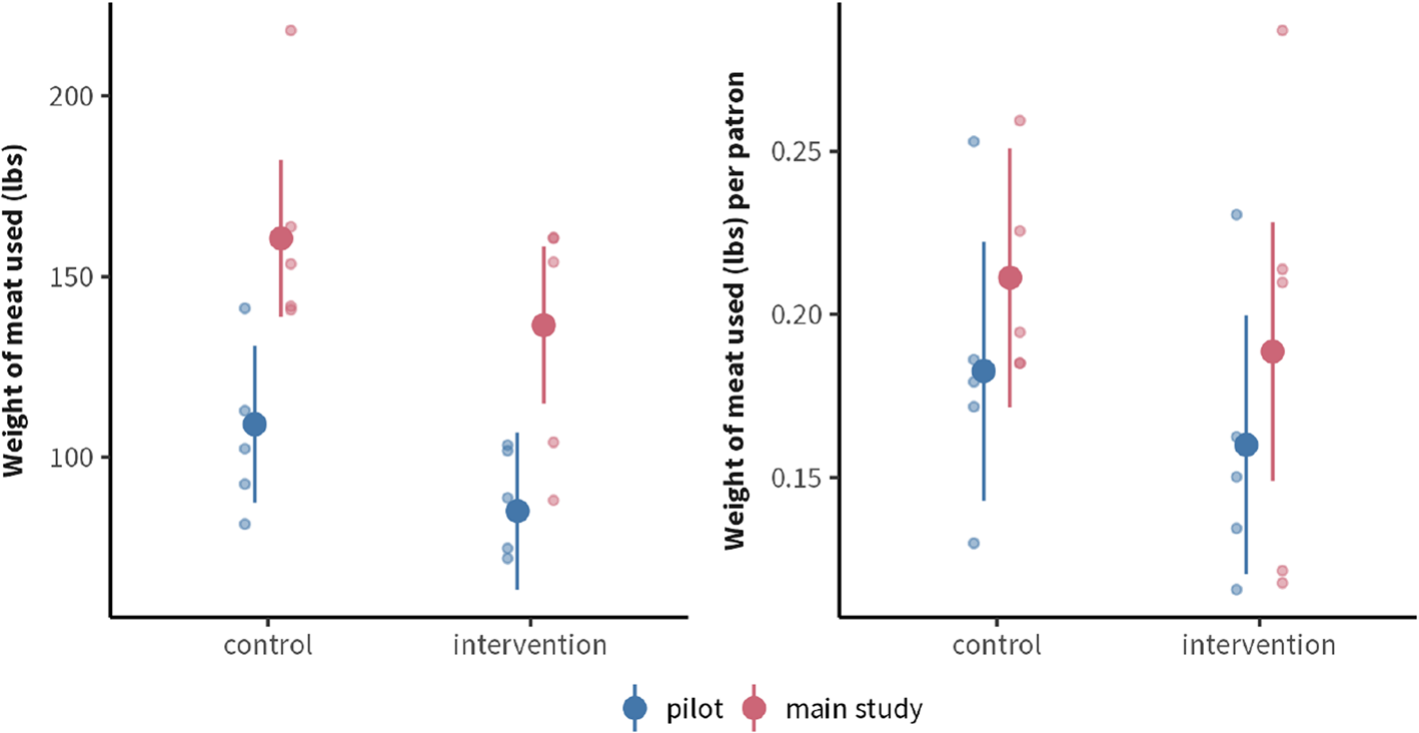
Total weight measures of meat served by staff from the intervention station at lunch. Small points represent individual days; large points represent means; bars represent 95% CIs. Left panel: the effect of the intervention vs. control condition. Right panel: secondary weight measure (i.e., normalized by the number of diners) showing considerably more variability

The estimated effect of the intervention was − 24.04 lbs (SE: 11.9, 95% CI: [− 49.2, 1.07], *p*-value: 0.059, Cohen’s d: 0.64), meaning around 24 lbs less meat was served in total on intervention days vs. control days (Fig. 4—left panel). Given the average of total meat served at the dining hall on control days (135 lbs), the estimated 24 lbs reduction is an 18% decrease in the amount of meat served. While this trend approached significance with a medium-to-large effect size, the confidence interval (CI) was wide and included the null. When normalized by the total number of diners at the dining hall, there was considerably more variability (Fig. 4—right panel). The effect was − 0.023 lbs/person (SE: 0.022, 95% CI: [− 0.065, 0.020], *p*-value: 0.27, Cohen’s d: 0.46), indicating no difference between conditions. We noted that more days of data collection were necessary to improve statistical precision, which motivated the implementation of Study 2.

#### Backfiring effects (H2)

**Fig. 5:**
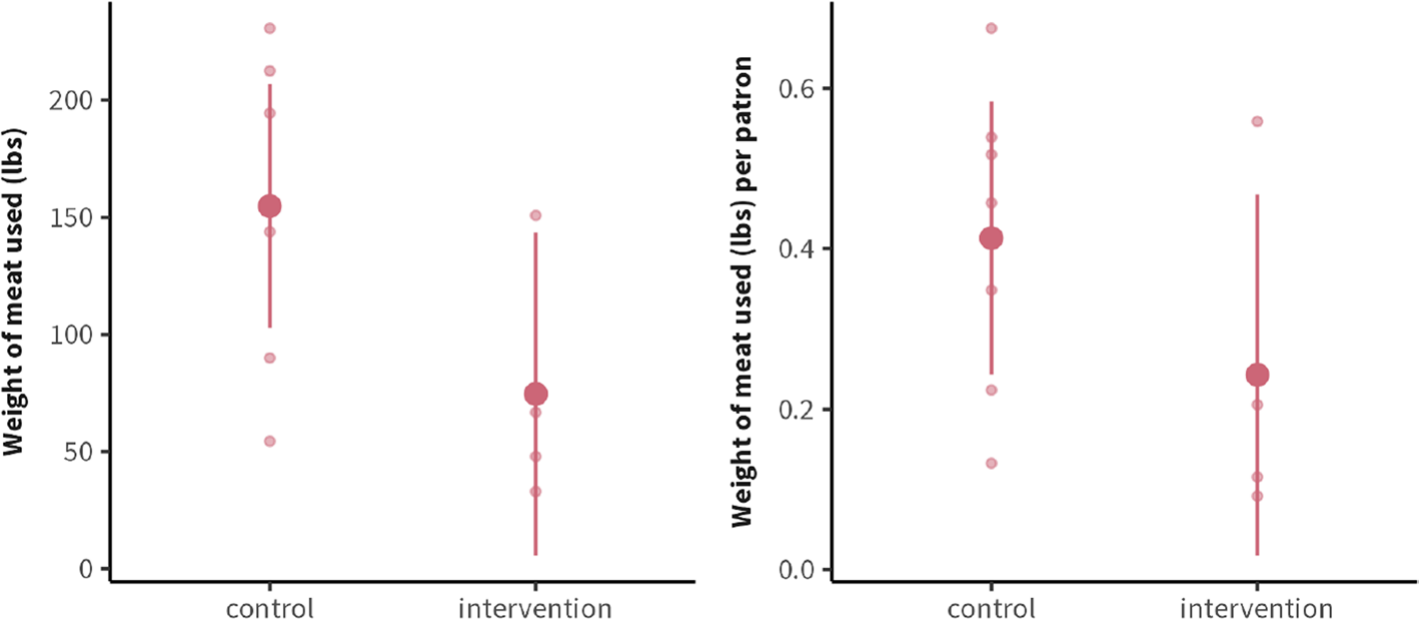
Total weight measures of meat served by staff at dinner across the entire dining hall (both intervention and control stations) to assess backfiring effects. Small points represent individual days; large points represent means, and bars represent 95% confidence intervals

We assessed potential backfiring effects at dinner as the intervention was only implemented during lunch.^4^ The total average meat consumption was 155 lbs on control days vs. 75 lbs on intervention days, indicating a reduction of 80 lbs (95% CI: [− 166.50, 6.14], *p*-value: 0.065) (Fig. 5—left panel). When normalized by the total number of diners, the effect is − 0.17 lbs/person (CI: [− 0.43, 0.089], *p*-value: 0.171), indicating no backfiring effects of the intervention at dinner (H2b). However, both weight measures had low precision in their effect estimations, necessitating more days of data collection.

#### Diner satisfaction (H3)

**Fig. 6:**
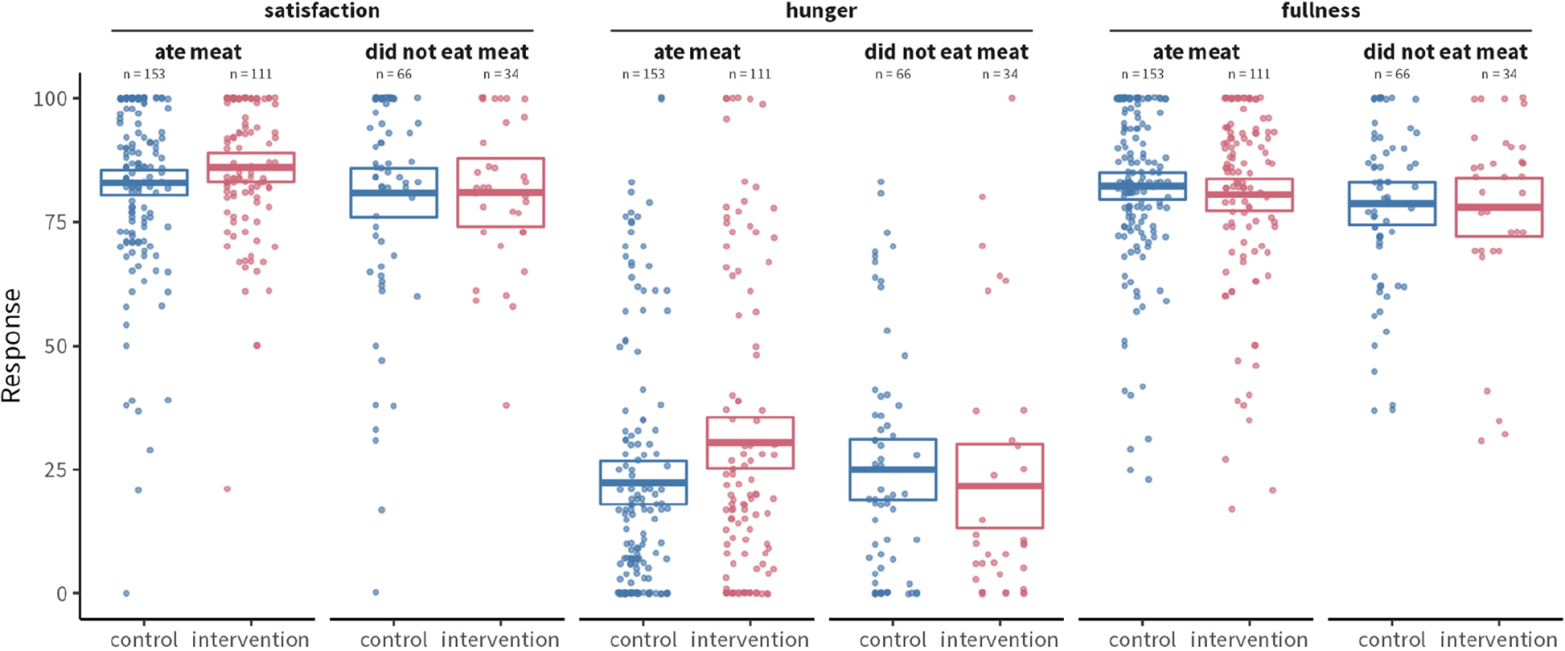
Survey respondents’ ratings of their satisfaction (left panel), hunger (middle panel), and fullness (right panel), separated by whether they ate meat as part of their meal. Boxes indicate means and 95% CIs

Across the three measures of satisfaction, hunger, and fullness, split by whether diners ate meat, the rating differences are very small, thus suggesting the intervention did not meaningfully detract from diners’ dining experience, especially in satisfaction and fullness (Fig. 6). The largest difference was in hunger for meat eaters, who reported a mean hunger level of 22.4 scale point during control (95% CI: [18.0, 26.8]) vs. 30.5 scale point during intervention (95% CI: [25.3, 35.6]). As such, participants in the intervention condition reported being significantly hungrier (*p*-value: 0.0195, Cohen’s d: 0.29) than participants in the control condition.

#### Controlling for meat types (H4)

When controlling for meat type (beef, pork, chicken, and fish), there was no effect of condition on satisfaction (*p*-value: 0.08), hunger (*p*-value: 0.12), and fullness (*p*-value: 0.52), with all coefficients near the null.

### Study 2: 50% spoon-size reduction with varying menu items

#### Demographics and sample size

A total of 29 days of data were collected for the weight measures, with 15 days (52%) under the intervention condition. The total number of diners eating at the dining hall was *n* = 6692 during lunch and *n* = 6063 during dinner. As the flyers specifically prompted survey responses from diners who ate at the intervention station, the resulting sample of *n* = 1802 (631 control/1171 intervention condition) represented 26.92% of all diners during the lunch period. More specifically, the weekly breakdown for survey responses was 153 (Week 1, control), 176 (Week 2, intervention), 200 (Week 3, control), 278 (Week 4, control), 494 (Week 5, intervention), and 501 (Week 6, intervention). Survey respondent demographics and dietary habits are presented in Table 1.

#### Meat consumption (H1)

**Fig. 7:**
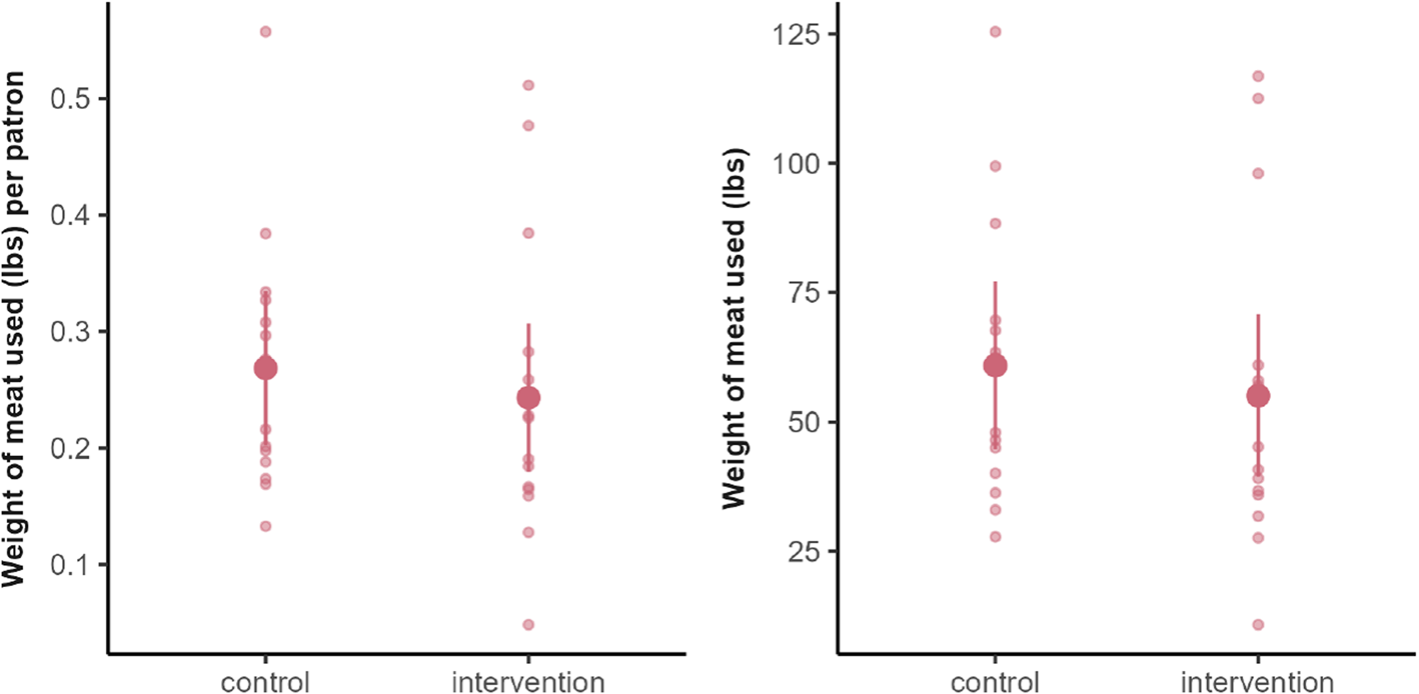
Distribution of meat served (lbs) on intervention vs. control weeks at the intervention station. Small points represent individual days; large points represent means, and bars represent 95% confidence intervals

The estimated effect of the intervention was − 5.82 lbs (95 CI: [− 28.35, 16.71], *p*-value: 0.60, Cohen’s d: 0.20), meaning around 6 lbs less meat was served in total on intervention days vs. control days, with a wide CI that overlapped the null. In the secondary weight measure, when normalized by the total number of diners at the dining hall, the effect was around − 0.03 lbs/person (95% CI: [− 0.12, 0.07], *p*-value: 0.58, Cohen’s d: 0.21), meaning on average 0.03 lbs less meat was served per person on intervention days (Fig. 7). Both results indicate there was no difference between conditions.

#### Backfiring effects (H2)

**Fig. 8:**
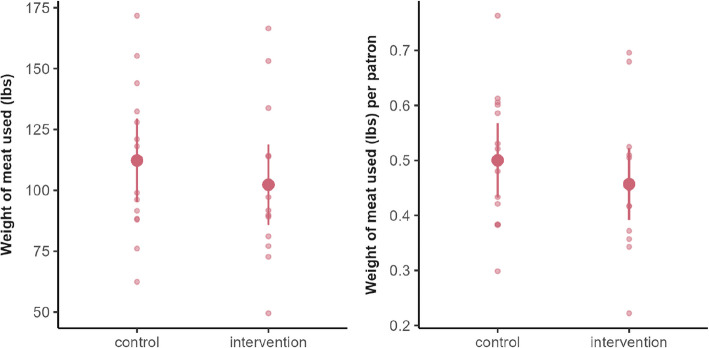
Distribution of meat served (lbs) on intervention vs. control weeks across the dining hall at lunch. Small points represent individual days; large points represent means, and bars represent 95% confidence intervals

The sample size for lunch was the same but one less day for dinner, thus 28 days of data were collected (50% under the intervention condition). During lunch (H2a), the estimated effect of the intervention was − 9.97 lbs (95% CI: [− 33.70, 13.76], *p*-value: 0.40, Cohen’s d: 0.32), meaning in total around 10 lbs less meat was served on intervention days vs. control days, but with a wide CI. On a per diner basis, this constitutes a non-significant reduction of 0.04 lbs/person (95% CI: [− 0.14, 0.05], *p*-value: 0.35, Cohen’s d: 0.35). Both results indicate there was no significant difference between conditions (Fig. 8).

**Fig. 9:**
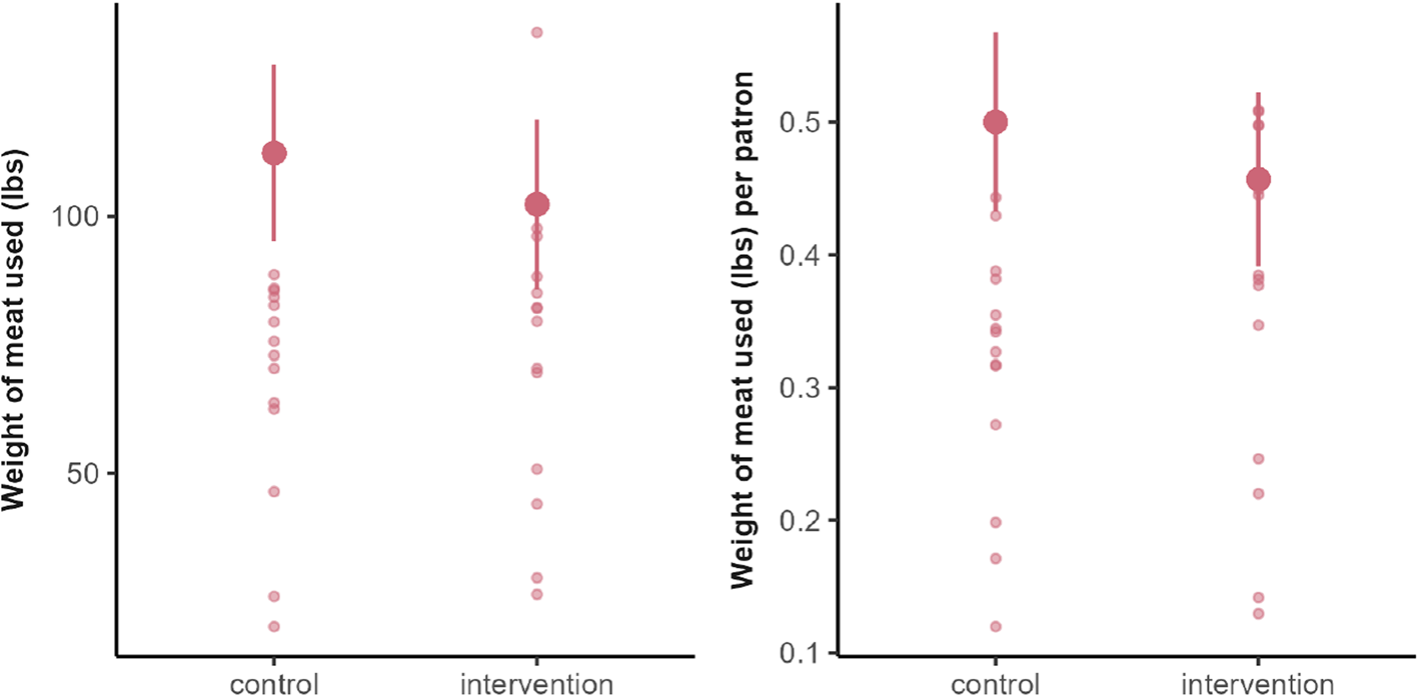
Distribution of meat self-served (lbs) on intervention vs. control weeks at dinner. Small points represent individual days; large points represent means, and bars represent 95% confidence intervals

During dinner, the estimated effect of the intervention was 6.67 lbs (95% CI: [− 13.41, 26.75], *p*-value: 0.50, Cohen’s d: 0.26), meaning around 7 lbs more meat was self-served in total on intervention days vs. control days, or 0.05 lbs/person (95% CI: [− 0.04, 0.14], *p*-value: 0.24, Cohen’s d: 0.45), also indicating no significant difference between conditions (Fig. 9).

##### Secondary analyses (H4)

We additionally controlled for meat type to reduce variation due to differences in popularity across meat protein types. This model also included interaction terms of meat type with intervention, although all coefficients were close to the null.

At lunch, the intervention appeared to reduce the amount of meat served by 49.60 lbs (95% CI: [− 119.96, 20.76], *p*-value: 0.16, Cohen’s d: 0.64), or 0.20 lbs/person (95% CI: [− 0.48, 0.06], *p*-value: 0.13, Cohen’s d: 0.69), at the intervention station, albeit with wide CIs. While non-significant, this estimated 50-lb reduction corresponds to a medium-to-large effect size. Across the entire dining hall (both intervention and control stations), the reduction was non-significant with a smaller effect size, at an estimated 24.55 lbs (95% CI: [− 97.32, 48.23], *p*-value: 0.50, Cohen’s d: 0.25), or 0.09 lbs/person (95% CI: [− 0.39, 0.20], *p*-value: 0.53, Cohen’s d: 0.24).

At dinner, however, the amount of meat self-served appeared to slightly increase on intervention weeks by 6.82 lbs (95% CI: − 28.79, 42.43], *p*-value: 0.70, Cohen’s d: 0.14), or by 0.07 lbs/person (95% CI: [− 0.11, 0.24], *p*-value: 0.46, Cohen’s d: 0.27). Given the wide CIs in main analysis and in this analysis, the reversal of effect direction is not meaningful.

When controlling for specific menu items at lunch at the intervention station, the intervention did not significantly reduce meat consumption either: the amount of meat served was reduced by 13.15 lbs (95% CI: [− 34.30, 7.99], *p*-value: 0.21, Cohen’s d: 0.60), though the CI was wide. Across the entire dining hall (both intervention and control stations), meat served at lunch decreased by 7.87 lbs (95% CI: [− 20.73, 5.00], *p*-value: 0.22, Cohen’s d: 0.42) but meat self-served at dinner increased by 16.77 lbs (95% CI: [− 13.53, 47.07], *p*-value: 0.26, Cohen’s d: 0.61), indicating a medium-to-large effect size with wide CIs.

Further analyses evaluated the relationship between the intervention and meat inclusion at the intervention station and found the intervention significantly increased the odds of participants reporting having eaten meat in their meal (OR = 1.08, 95% CI: [1.04. 1.13], *p*-value: < 0.001).

#### Diner satisfaction (H3)

Among 1802 completed surveys, 1560 participants indicated having eaten at the intervention station (*n* = 1,026 or 66% during intervention weeks). The primary outcome of interest was satisfaction, which on intervention vs. control days was − 6.72 points (95% CI: [− 9.47, − 3.97], *p*-value: < 0.001, Cohen’s d: 0.26), meaning overall diner satisfaction significantly decreased on intervention days. The effect of intervention vs. control on fullness was similar, leading to a significant decrease in fullness by 7.45 points (95% CI: [− 10.17, − 4.74], *p*-value: < 0.001, Cohen’s d: 0.29). Complementing the decrease in satisfaction and fullness, hunger increased by 6.81 points (95% CI: [4.00, 9.61], *p*-value: < 0.001, Cohen’s d: 0.25), indicating diners reported feeling significantly hungrier on intervention vs. control days. When split by whether participants ate meat, the difference in satisfaction is slightly greater between intervention vs. control days. Among 1280 responses reporting meat consumption, we found significant decreases in overall satisfaction by 9.25 points (95% CI: [− 9.47, − 3.97], *p*-value: < 0.001, Cohen’s d: 0.34) and fullness by 9.53 points (95% CI: [− 10.17, − 4.74], *p*-value: < 0.001, Cohen’s d: 0.36), and a significant increase in hunger by 8.61 points (95% CI: [4.00, 9.61], *p*-value: < 0.001, Cohen’s d: 0.32).

#### Controlling for meat types (H4)

When controlling for meat type, the intervention did not meaningfully affect overall satisfaction, given differences of only 1.02 points (95% CI: [− 7.94, 9.97], *p*-value: 0.82, Cohen’s d: 0.01) and of 0.93 points for fullness (95% CI: [− 7.94, 9.79], *p*-value: 0.84, Cohen’s d: 0.01). However, unexpectedly, the intervention significantly decreased hunger by 10.57 points (95% CI: [− 19.66, − 1.48], *p*-value: 0.02, Cohen’s d: 0.12). Meat type significantly decreased satisfaction when comparing pork to beef by 14.65 points (*p*-value: 0.02), and significantly decreased fullness when comparing chicken, fish, and pork to beef, by 9.03 points (*p*-value: 0.06), 12.01 points (*p*-value: 0.06), and 10.63 points (*p*-value: 0.08), respectively. Thus, meat type significantly modified how hungry diners were on intervention vs. control days across all meat types, with diners reporting significantly lower levels of hunger following beef consumption compared to all other meat protein sources (*p*-value: < 0.001).

### Total meat consumption and diner satisfaction: Aggregate data

Combining Study 1 and Study 2, there was no significant difference between conditions. Although the trend indicated a reduction in the total amount of meat served during the intervention as − 12.05 lbs (95% CI: [− 31.90, 7.79], *p*-value: 0.22, SMD: 0.24), or—0.02 lbs/person (95% CI: [− 0.09, 0.05], *p*-value: 0.49, SMD: 0.22), when controlling for meat type, the trend reversed and indicated an increase in meat served by 12.25 lbs (95% CI: [− 75.71, 100.21], *p*-value: 0.78, SMD: 0.24), or 0.02 lbs/person (95% CI: [− 0.27, 0.32], *p*-value: 0.86, SMD: 0.23). Given these wide CIs, the reversal of effect direction is not meaningful.

## Discussion

The primary hypothesis (H1), namely that less meat will be served, and by proxy consumed, at the intervention station during intervention periods was not supported across the two studies. The apparent difference in the total daily amount of meat served in Study 1 (~ 24 lbs reduction) and Study 2 (~ 6 lbs reduction) may be explained by several factors, many of which highlight commonly observed challenges in large-scale, real-life interventions [27, 52]. These include variations in the application contexts, such as differences in meal types (i.e., burritos vs. varying menu items) and unintended backfiring effects in Study 2.

Firstly, we suggest the 3 oz serving spoon (25% reduction) in Study 1 was less visually salient to diners than the 2 oz spoon (50% reduction) in Study 2 (Fig. 2b). The latter may explain why diners in Study 2 were paradoxically more likely to report having eaten meat, since some diners (e.g., flexitarians) may only choose to consume meat when the portion size is small. According to anecdotal reports by the serving staff, diners in Study 2 often asked for a second serving of the meat during intervention periods, which would cancel out its effects. Most participants were undergraduate students living in the university’s neighborhood system, many of whom are regular diners at Dining Hall 2. As a result, they may have noticed the default portion sizes being halved during the intervention periods and requested a second serving. Indeed, we found that the intervention significantly increased the odds of participants reporting having eaten meat in their meal (OR = 1.08, 95% CI: [1.04. 1.13], *p*-value: < 0.01). In Study 1, the meat portion in the burritos was immediately concealed when staff wrapped the tortilla, but in Study 2, menu items often lacked such concealment, making the reduced meat portion size more noticeable.

While default nudges are generally considered the most potent behavior change levers [53], likely due to minimizing the diner’s cognitive load [27], their range of effectiveness is nonetheless limited. Our findings align with the ‘norm range model’ [44], which suggests that moderate, but not drastic, portion size reductions may decrease consumption without triggering compensatory eating behaviors. The smaller reduction in Study 1’s spoon size (25%), which is more likely to fall within the model’s perceived normal range [44], followed this trend but still did not lead to a significant decrease in the amount of meat served. Further research is needed to continue identifying the boundaries and limits of this ‘norm range’ in real-life food settings in order to design more effective behavioral interventions that avoid backfiring effects.

We found more variation than anticipated in the daily number of diners in Study 2, likely because some menu items were more popular than others (e.g., the “Korean fried chicken” dish was the most popular and attracted the most diners to the intervention station). This factor was not present in Study 1, where burritos were the only menu item. In Study 2, our chef and dining hall partners reported that the intervention station almost always sold out, likely due to the ‘lunch special’, which appeared to attract and sway diners who otherwise might not have consumed meat at that station (H2a), or meat that day in general, or even lunch at this dining hall. Kitchen staff also noted that diners who typically would not return to this neighborhood for lunch seemed more likely to do so because of the ‘lunch special’. A large white board, which advertised this ‘special’ in front of the dining hall (Fig. 2b), may have unintentionally primed and attracted diners specifically to the intervention station, potentially contributing to additional variation in the data. Furthermore, due to the popularity of the intervention’s ‘lunch special’, the non-intervention station had more leftover meat that was repurposed into a ‘dinner special’. This also attracted more diners, thereby increasing meat consumption at dinner and contributing to backfiring effects (H2b). While these forms of variation are inherent in real-life food services, they reduce statistical precision and thus power to detect intervention effects.

Regarding diner satisfaction (H3), the observed differences between the two studies may reflect practical differences in serving sizes of the overall dish. Specifically, Study 1’s burritos were particularly large and thus likely more filling regardless of the meat portion size. This may have helped offset the reduced portion sizes of meat during intervention, resulting in no significant differences in diner satisfaction, fullness, and hunger. In contrast, Study 2’s varying menu items were potentially less filling, which, alongside the more drastic meat portion size reduction, may explain the significant decreases in satisfaction and fullness and increase in hunger. This underscores the importance of not only reducing the meat portion size but also increasing the non-meat protein and/or plant-based portion sizes to compensate for the difference in future studies. Indeed, Reinders and colleagues (2017) reduced the meat and fish portion size by 12.5% but also increased the plant portion size by 100%, resulting in 11% smaller meat and fish consumption, 31% more vegetable consumption, and no difference in diner satisfaction [41]. While increased vegetable intake has long been recommended to offset reduced volume and enhance satiation, sudden reductions in overall volume or energy content have been shown to lead to undesirable compensatory behaviors [47, 48].

Our finding that diners reported feeling the least hungry after consuming beef compared to all other meat types (H4) is consistent with its second-highest ranking among protein-rich foods on the Satiety Index [54]. As protein is the most satiating macronutrient [55], future research should investigate the potential role that plant-based protein can play in offsetting negative effects on diner hunger in such nudge interventions. As for the unexpected finding in Study 2 that, when controlling for meat types, the intervention significantly decreased hunger, we suspect this is likely spurious given the low R-squared value (0.03329) and cannot confidently explain its occurrence without further investigation.

Regarding strengths and limitations, the strengths of this research are its rigorous methodology and real-life setting that offer value insights for similar food service models across the industry, including cafeterias, school canteens, and staff-served restaurant chains. We successfully measured and provided insights into backfiring effects and the determinants of diner satisfaction, both of which have been substantial barriers to the broader scalability and adoption of such interventions by the food service industry [56]. Limitations include the lack of data on survey response rates specific to the intervention station’s diners and the relatively small number of data collection days that, especially in Study 1, decreased precision. Another limitation is the lack of long-term mitigation of diners’ environmental footprints, as this default nudge can only change behavior at the specific time and place of its implementation [57]. Our measurement of backfiring effects is somewhat limited due to the lack of data on the specific study sample at lunch versus dinner; however, the university’s neighborhood system suggests a high degree of overlap between meal periods. Given our specific goal of reducing meat consumption, we did not measure consumption of non-meat items but suggest this for future research. We used meat served as a proxy for meat consumption and did not assess leftover meat that diners returned or discarded as waste but also recommend this for future research. Finally, we lacked sufficient data collection days and menu items to analyze the impact of the more detailed characteristics of menu items. Future studies should focus on mapping the situational and contextual ‘norm range’ of default portion size nudges to reduce meat consumption across different menu items and food service models.

## Conclusion

Our findings are consistent with the null to medium effect sizes—depending on publication bias adjustment—commonly reported for behavioral nudge interventions [27, 45]. While taken together the two interventions (25% and 50% serving spoon size reductions) did not significantly reduce the amount of meat served, the daily decrease of ~ 12 lbs with a small effect size (SMD = 0.24) showed a trend in the expected direction. Differences in the results of Study 1 and Study 2 highlight the nuanced challenges of finding the ‘norm range’ boundaries of portion size interventions in real-life settings. The medium effect sizes in Study 1, alongside the absence of meaningful negative impacts on overall diner satisfaction, suggest higher likelihoods of intervention success when the reduced meat portion size is concealed and/or offset by the overall meal’s relatively larger portion size, as is the case with burritos (e.g., sandwiches, paninis, calzones, wraps, empanadas, tamales, quesadillas, etc.). The small effect sizes, backfiring effects, and significantly decreased diner satisfaction in Study 2 suggest that a drastic serving size reduction likely violates the ‘norm range’, particularly in contexts where regular diners are more likely to notice such changes. In contrast to the large and filling burritos in Study 1, the greater variety of menu items in Study 2 highlight the importance of offsetting any differences in protein and meal portion sizes with plant-forward and plant-based options. These findings offer valuable insights for scaling cost-effective and easy-to-implement meat reduction strategies across various high-volume food service settings, including both commercial establishments (e.g., American chain restaurants such as Chipotle, Subway, and poke bowl bars) and noncommercial ones (e.g., K-12 schools, healthcare facilities, and corporate dining).

## Data Availability

All data, code, and materials required to reproduce our results are publicly available on the Open Science Framework (OSF), as described in the manuscript.

## Footnotes

1 The survey questionnaire is available at https://osf.io/2hy74/.

2 Available at https://osf.io/2hy74/registrations.

3 Our pre-registration stated that internal meta-analyses would be conducted while including interaction terms of condition with study. It is possible to obtain the average effect between studies from this model, but this coincides with simply omitting the interaction term, so we opted for the latter approach.

4 Results of the post hoc analysis (H2a): During lunch, the estimated effect of the intervention was - 25 lbs (95% CI: [- 79, 29], *p*-value: 0.34, Cohen’s d: 0.44), meaning in total 25 lbs less meat was served on intervention days vs. control days, but with a wide CI. On a per diner basis, this constitutes a non-significant reduction of 0.02 lbs/person (95% CI: [- 0.097, 0.056], *p*-value: 0.35, Cohen’s d: 0.25), thus indicating no backfiring effects of the intervention at lunch.
